# Current Evidence Using Pulsed Electromagnetic Fields in Osteoarthritis: A Systematic Review

**DOI:** 10.3390/jcm13071959

**Published:** 2024-03-28

**Authors:** Luigi Cianni, Emidio Di Gialleonardo, Donato Coppola, Giacomo Capece, Eugenio Libutti, Massimiliano Nannerini, Giulio Maccauro, Raffaele Vitiello

**Affiliations:** 1 Orthopaedics and Trauma Surgery Unit, Catholic University of the Sacred Heart, 00168 Rome, Italy; luigi_cianni@libero.it (L.C.); emidiodiggia@gmail.com (E.D.G.); donato.coppola07@gmail.com (D.C.); giulio.maccauro@unicatt.it (G.M.); lele.vitiello@gmail.com (R.V.); 2 Department of Ageing, Neurosciences, Head-Neck and Orthopedics Sciences, Orthopedics and Trauma Surgery Unit, Fondazione Policlinico Universitario Agostino Gemelli IRCCS, 00168 Rome, Italy; 3 Aurelia Hospital, 00165 Rome, Italy; elibutti@alice.it (E.L.); massinannerini@gmail.com (M.N.)

**Keywords:** pulsed electromagnetic fields, musculoskeletal disorders, osteoarthritis, application, therapy

## Abstract

(1) **Background:** Osteoarthritis (OA) significantly impacts patients’ quality of life and negatively affects public healthcare costs. The aim of this systematic review is to identify the effectiveness of pulsed electromagnetic fields (PEMFs) in OA treatment across different anatomical districts, determining pain reduction and overall improvement in the patient’s quality of life. (2) **Methods:** In this systematic review following PRISMA guidelines, PubMed and Google Scholar were searched for randomized controlled trials involving patients with osteoarthritis undergoing PEMF therapy. Seventeen studies (1197 patients) were included. (3) **Results:** PEMF therapy demonstrated positive outcomes across various anatomical districts, primarily in knee osteoarthritis. Pain reduction, assessed through VAS and WOMAC scores, showed significant improvement (60% decrease in VAS, 42% improvement in WOMAC). The treatment duration varied (15 to 90 days), with diverse PEMF devices used. Secondary outcomes included improvements in quality of life, reduced medication usage, and enhanced physical function. (4) **Conclusions:** Diverse PEMF applications revealed promising results, emphasizing pain reduction and improvement in the quality of life of patients. The variability in the treatment duration and device types calls for further investigation. This review informs future research directions and potential advancements in optimizing PEMF therapies for diverse osteoarthritic manifestations.

## 1. Introduction

### 1.1. Definition and Classification

Osteoarthritis (OA) is a degenerative process primarily involving the articular cartilage, with secondary damage to the subchondral bone and synovial membranes [1].

OA can be classified as primary (idiopathic) or secondary (more frequently post-trauma or post-surgical) [2]. It is a condition that not only greatly impacts the quality of life of patients, but also has an effect on the healthcare system because of the costs related to its management [3]. It is clear from the literature that osteoarthritis is not only a condition confined to the musculoskeletal system but is also related to the onset of cardio-vascular and mental disorders [3,4,5,6].

Cartilage degeneration can be assessed using the Outerbridge Classification [7], which was developed in 1961 and is based on the direct visualization of cartilage, either arthroscopically or open, assigning a severity grade ranging from 0 (normal cartilage) to 4 (subchondral bone exposure) [8]. Although the Outerbridge Classification is widely used in the clinical field, it has many limitations, such as poor reproducibility among surgeons and no correlation with prognosis or type of treatment [8].

### 1.2. Epidemiology

The prevalence of OA in the entire population is about 15%, with an increasing trend in future years [9]. The female sex is more affected than the male sex, with a worse radiologic pattern [9]. According to the literature, in North American and European populations, the district most affected by osteoarthritis is the hand (66%), followed by the knee (33%) and the hip (5%) [2].

### 1.3. Risk Factors

In the literature, there are studies that show that in the population, osteoarthritis affects a specific group of people, depending on whether they perform a specific type of work or follow a specific lifestyle (exercise; diet). Furthermore, people with certain genes that promote the production of pro-inflammatory cytokines and lytic enzymes have a higher incidence of osteoarthritis [10,11,12].

Many risk factors are implicated in the occurrence of OA, such as age, genetic factors, malalignments and joint trauma, occupation and diet [10,11,13].

A study by Perry et al. found that occupation plays a major role in the onset of osteoarthritis, with heavy manual labor in particular increasing the incidence of knee osteoarthritis in men [11].

Justin et al., in their review and meta-analysis, show how there is a close correlation between high BMI and the onset of OA, as well as a protective effect of serum calcium and a weak protective effect of low-density lipoprotein (LDL) levels on the onset of OA [14].

### 1.4. Sign and Symptoms

Pain is the most prominent clinical manifestation, and is closely related to the anatomical district affected, with the hip and knee being most involved in the genesis of painful symptoms [2].

The clinical presentation is characterized by a decreased passive and active range of motion, stiffness, and, in severe cases, joint deformities [15].

### 1.5. Diagnostic Methods and Treatment Options

Radiological investigations play a predominant role in the diagnosis of OA. In 1957, Kallegren and Lawrence created a radiological classification system that is frequently applied to the knee joint. It is a system that, starting from radiological images, assigns a severity grade ranging from 0 (no OA) to 4 (severe OA) [16].

There are several alternatives for the treatment of osteoarthritis, all focused on reducing the symptoms related to this condition [15]. The first line of treatment is the reduction in risk factors such as body weight; there is evidence in the literature that body weight loss reduces stress on the joints, preventing the onset and progression of the disease [15]. Pharmacological treatment is reserved for patients who have not responded to the first line of treatment and consists of the administration of non-steroidal anti-inflammatory drugs, intra-articular infiltration and the administration of biological drugs [17]. Prosthetic joint replacement should be considered when there is a failure of conservative therapy and a severe radiographic picture of OA [18]. 

In the literature, many studies have evaluated the effectiveness of pulsed electromagnetic fields (PEMFs) in the treatment of OA. PEMFs were introduced into clinical practice in 1970 and their effectiveness in the treatment of nonunion fracture has been widely demonstrated [19,20].

PEMFs use frequencies ranging from 6 to 500 Hz that can stimulate currents in tissues with biological effects [21]. Animal studies have shown that the use of PEMFs can reduce the progression of OA by inhibiting TNF alpha and IL-6 signaling [22]. Several in vitro studies have aimed to demonstrate the mechanism of action of PEMFs. Petecchia et al. demonstrated the role of PEMFs in the osteogenic differentiation of human mesenchymal stem cells derived from bone marrow stroma, modulating the cytosolic concentration of calcium and the expression of voltage-gated calcium channels [23]. Brighton, C T et al. also demonstrated the role of calcium as a key element in the intracellular transduction of the signal produced by electromagnetic fields [24]. PEMFs are involved in the expression of genes that promote osteogenic cell differentiation and the production and mineralization of the extracellular matrix [25]. At the level of the cell membrane, PEMFs, being too weak, fail to generate membrane depolarization, but are involved in amplifying the trans-membrane signal by favoring the binding of the ligand to its receptor, triggering intra-cellular mechanisms involved in osteogenesis, cell proliferation and differentiation and immune modulation [26].

Randomized controlled studies in the literature concerning the efficacy of PEMFs in the treatment of osteoarthritis have shown conflicting results [27,28]. Markovic et al., in a systematic review of systematic reviews, showed that the use of PEMFs has a positive effect on disability and physical function of patients with osteoarthritis in only 5 out of 10 studies analyzed, while in 1 study, no statistically significant effect of PEMFs was reported [29]. This reflects the discordance in the literature concerning the use of PEMFs in the treatment of osteoarthritis. The variability in results could derive from the different durations of treatment with PEMFs in the various studies and the different frequency of weekly use, and also the application of different PEMF devices. The use of additional therapies for osteoarthritis at the same time as PEMFs may mask the efficacy of the latter.

### 1.6. Study Objectives

In this review, we analyzed various studies in the literature with the aim of assessing the effectiveness of PEMFs in the treatment of OA in different anatomical districts and their ability to improve patients’ quality of life by acting on various outcomes such as pain reduction.

## 2. Methods

Standard systematic review methods were used. The literature search was performed by three of the authors (GC, EDG and DC), independently of each other. This meta-analysis was performed based on the PRISMA (Preferred Reporting Items for Systematic Reviews and Meta-Analyses) guidelines, ensuring a comprehensive and systematic approach to data retrieval and synthesis. This systematic review has been appropriately registered with the International Prospective Register of Systematic Reviews (PROSPERO) under registration number 524542.

Two electronic databases (PubMed and Google scholars) were searched for randomized controlled trials (RCTs) through using following the combination of a series of keywords and text terms: “arthritis”, “osteoarthritis”, “arthropathy”, “PEMF”, “Pulsed ElectroMagnetic Fields”. The exact search string used was ((arthritis[Title/Abstract]) OR (osteoarthritis[Title/Abstract]) OR (arthropathy[Title/Abstract])) AND ((PEMF[Title/Abstract]) OR (Pulsed ElectroMagnetic Fields[Title/Abstract])).

The literature references of identified papers were also searched in order to find further relevant articles. All journals were considered.

**Figure 1:**
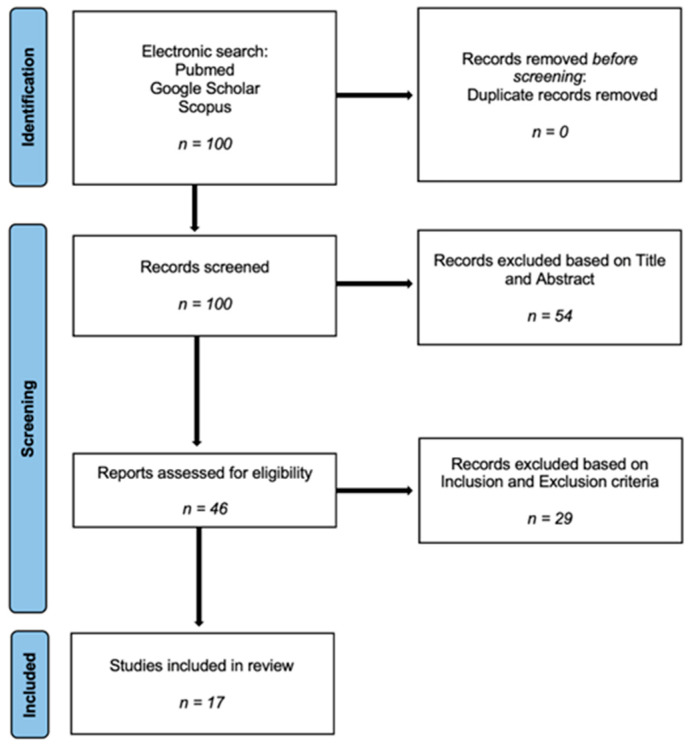
PRISMA flowchart.

Our search strategy aimed to identify randomized controlled trials (RCTs) relevant to the therapeutic application of PEMFs in osteoarthritis. To minimize the number of missed studies, no time restrictions and no filters were applied to the search strategies. Article titles and abstracts were reviewed and the articles of interest were selected for the full text. The bibliography of the selected studies was accurately searched by hand to identify further studies not found during our electronic search. No restrictions on the date of publication or language were applied. After conducting a literature search, 100 papers were selected for further evaluation. Of these, 54 were excluded because the title or abstract did not meet the inclusion criteria. Of the remaining studies, through the analysis of the materials and methods, 29 were excluded because they were designed as experimental studies (in vitro studies, animal studies or cadaveric studies) or related to other inflammatory focuses (tendinitis, peri-implantitis). Ultimately, the 17 remaining papers met all of the criteria [Figure 1].

The extracted data stemmed from scientific studies published from 2001 to 2023. Of these studies, 94.12% were randomized clinical trials.

### 2.1. Inclusion and Exclusion Criteria

The eligibility criteria for inclusion in our meta-analysis were set to ensure the selection of studies meeting rigorous standards. Studies were included if they were designed as an RCT and the subjects had osteoarthritis (OA) of one or multiple joints and underwent PEMF therapy alone or in combination with other therapeutic modalities (surgery, infiltrative therapy with cortisone and/or hyaluronic acid).

Exclusion criteria were studies designed as a systematic review, meta-analysis, or experimental study (in vitro studies, animal studies or cadaveric studies) or had another inflammatory focus (tendinitis, etc.). The three reviewers (GC, EDG and DC) evaluated the full text of the selected articles to determine whether they were eligible for inclusion and collected data of interest. In case of doubt regarding the inclusion of an article, the senior author made the final decision.

The three authors (GC, EDG and DC) independently assessed the risk of bias. A supervisor [LC] was consulted in case of disagreement.

### 2.2. Data Extraction and Outcome Measure

Detailed information was systematically extracted from each selected study. Three authors (GC, EDG and DC) extracted the data in a predefined excel sheet. The extracted information included the number and gender of participants in each trial, the treatment protocol of the PEMFs, the anatomical site of PEMF application, description and duration of the application and the type of outcome measures. The main goal of this study was to evaluate the efficacy of pain alleviation and function improvement by applying PEMF therapy for patients with OA. The Western Ontario and McMaster Universities Osteoarthritis Index (WOMAC) function, the VAS score, and the Knee Injury and Osteoarthritis Outcome Score (KOOS) were the preferred measures for pain and function outcome.

## 3. Results

The encouraging results emerging from the various analyzed studies collectively contribute to the understanding of the role of PEMF therapy in osteoarthritis management, highlighting its diverse applications and potential to improve patients’ clinical outcomes.

The cumulative analysis of the reviewed studies provides valuable insights into the application of PEMF therapy in osteoarthritis, considering key parameters such as the total number of enrolled patients, the involved anatomical districts, and different types of PEMF utilized.

### 3.1. Demographics

Through our literature review, we analyzed 17 scientific studies, comprising a total of 1197 patients with osteoarthritis affecting various anatomical districts. In 8 out of 17 scientific studies (47.1%), the gender of the enrolled patients was not specified. However, among the 332 enrolled patients for whom gender was specified, 119 were men (35.8%), and 213 are women (64.1%) [Table 1]. 

We observed a diversified range in the studies, with sample sizes ranging from 22 to 428 individuals. This variability underscores efforts to explore the impact of PEMFs on different cohorts, ensuring a comprehensive understanding of its effectiveness. Larger studies, such as that conducted by Xiang et al. [30], including 428 participants, contributed considerable statistical power to assessing the non-inferiority of PEMFs compared to, for example, COXIB therapy in managing knee osteoarthritis.

### 3.2. Type of PEMF Device

These studies investigated the utilization of both presently available PEMF devices on the market and outdated, no longer used equipment. The variety of PEMF devices employed in these studies introduced an additional layer of complexity to the analysis. [Table 2]. Devices such as those from the Bioelectronics Corporation (BioElectronics Corporation- Metropolitan Court Fredrick, MD, USA), Magcell Arthro (Physiomed Elektromedizin-Hutweide, Schnaittach, Germany), and XT-2000 B by the Better Health Corporation (Abbotsford, Victoria, Australia), each characterized by unique specifications like the frequency and intensity of the magnetic field, highlight the technological variability within PEMF therapy. For instance, the Bioelectronics Corporation’s device has a carrier frequency of 27.12 MHz and emits 1000 pulses per second. On the other hand, Magcell Arthro emits pulsed magnetic fields at a high power reaching up to 100 mT (1000 gauss), with a selective frequency range. The study also explores the utilization of I-ONE by IGEA Medical (IGEA SpA, Carpi, Italy), featuring a frequency of 75 Hz and a magnetic field intensity ranging from 10 to 18 Gauss at its peak. On the other hand, Elettronica Pagani’s device (Elettronica Pagani Srl, Paderno Dugnano, Italy) has a frequency of 1–3 MHz with both continuous and pulsed emission (ranging from 20% to 80%), while Fisiofield (Fisioline Srl, Borgata Molino, Italy) provides a notably high field intensity, exceeding 100 Gauss at its peak for each applicator.

### 3.3. Treatment Duration

The average duration of PEMF application ranged from 15 days to 90 days (median 52.5) [Table 1]. Short-term interventions, such as the two-week trial by Ozgüçlü et al. [28] and the 18-day treatment in Wuschech et al. [31], aimed to assess more immediate effects. Conversely, long-term studies, like the 90-day intervention implemented by Zorzi et al. [32], were designed to evaluate the sustainability and cumulative impact of PEMF therapy on functional recovery after arthroscopic surgery. Short-term treatments showed a notable reduction in acute pain, while long-term interventions were effective in preventing exacerbations and promoting sustained benefits. The studies analyzed had treatment frequencies ranging from every 10 min to every 12 h each day, and it is evident that a higher treatment frequency showed more encouraging results.

### 3.4. Anatomical Districts Involved

Thirteen studies (77%) focused on knee osteoarthritis, one study focused on the lumbar spine [33], one focused on the cervical spine [34], one focused on osteoarthritis of the lower limbs in general, and a final study evaluated both knee osteoarthritis and cervical spine osteoarthritis [Table 1]. One of these studies also assessed conditions such as hemophilic arthropathy in the knee joint [35], expanding the scope of potential applications of PEMF therapy.

In patients with knee osteoarthritis, the extent of the condition was predominantly evaluated using the Kellgren–Lawrence (KL) classification. Specifically, this classification was employed in six articles (35.2%) to determine the severity of arthritis. Among the scientific articles, five (71%) studied moderate arthritis (grade 2–3), while one article (14.2%) focused on a milder form of arthritis (grade 0–2). In a solitary study (14.2%) dedicated to knee osteoarthritis, the American College of Rheumatology criteria were utilized.

### 3.5. Outcomes

The analysis of results from PEMF therapy studies consistently highlighted pain reduction as the primary focus in most studies. The reduction in pain intensity was often measured using tools like visual analog scales (VAS) or disease-specific scales such as the Western Ontario and McMaster Universities Osteoarthritis Index (WOMAC). Positive trends in the analgesic effects of PEMFs were consistently reported, with significant improvements in pain relief across various studies. Secondary outcomes varied, reflecting the multifaceted impact of PEMF therapy on osteoarthritis. These included assessments of quality of life, changes in analgesic and non-steroidal anti-inflammatory drug (NSAID) intake, improvements in physical function, and severity assessments of the disease. For instance, the studies by Bagnato et al. [27] and Dündar et al. [36] delved beyond pain reduction, exploring improvements in quality of life and the utility of specific biomarkers like YKL-40 in assessing disease severity and treatment response.

Taking a closer look at studies with encouraging results, in five (29.4%) of the analyzed scientific studies, the VAS scale was used to assess the effects of PEMF therapy in patients with osteoarthritis, showing a significant 60 ± 11% decrease in VAS pain scores [Table 1].

As for the WOMAC global score, which was employed in six studies (35.2%) among those analyzed, it revealed a notable average improvement of 42% (95% CI −85 to 17) [Table 1]. Additionally, a substantial reduction in knee-related and WOMAC pain scores (8.5 ± 0.4) with PEMF therapy was observed, along with enhancement in the WOMAC disability score.

Furthermore, other assessment scales, utilized in 5.8% of the analyzed studies, such as the EuroQol score, KOOS, and Neck Pain and Disability Scale (NPDS) score, showcased highly favorable results in the PEMF group.

**Table 2:** Main features and outcomes of the studies included in the research.

	Research Study	Number of Patients	Type of PEMF Device	Treatment Duration	Anatomical District	Assessment Scales	Outcomes
1	Bagnato et al. (2016) [27]	60 (43 F, 17 M)	Bioelectronics Corporation (27.12 MHz)	12 h per day for 1 month	Knee	WOMAC, VAS, SF-36	Pain reduction (primary outcome), assessment of quality of life (secondary outcome)
2	Wuschech et al. (2015) [31]	57	Magcell Arthro	18 days (twice a day for 5 min)	Knee	WOMAC, VAS	Pain reduction, stiffness, and disability; tolerability and efficacy
3	HF Liu et al. (2015) [33]	50 (F)	XT-2000 B	5 weeks	Spine	Vas, SF-36, ODI; MMT score; BBS score; TUGscore	Primary outcome (change in femur bone mineral density); secondary outcome (change in mineral bone density of lumbar spine)
4	Dündar et al. (2016) [36]	40		4 weeks	Knee	VAS, WOMAC	Pain reduction; utility of YKL-40 in assessing the severity of the condition
5	Gobbi A et al. (2014) [20]	22 (11 M; 11 F)	I-ONE IGEA	45 days (4 h per day)	Knee	VAS, IKDC objective (KOOS); Tegner score	ROM, pain relief, improvement of symptoms, and improvement of activity level
6	Iannitti T et al. (2013) [37]	28	F&B International	6 weeks	knee	VAS, WOMAC	Pain relief, stiffness, physical function
7	Nelson et al. (2012) [38]	34 (24 F; 10 M)			knee	VAS	Pain relief
8	Ozgüçlü et al. (2010) [28]	40 (29 F; 11 M)	Device Elettronica Pagani	2 weeks	knee	VAS, WOMAC	Greater effectiveness than other non-surgical treatments
9	Khami et al. (2020) [35]	40 (M)	Fisiofield Maxi	18 sessions (3 times a week for 6 weeks)	Knee	Pettersson radiographic criteria, clinical signs, QoL, VAS, HJHS	The application of PEMFs could help to prevent further joint damage and prevent functional decline in patients
10	Xiang et al. (2022) [30]	428	Better Health Corporation (15 Hz, 30 mT)	6 weeks (40 min/day, 5 days a week)	Knee	WOMAC pain index, WOMAC function and stiffness, pain, quality of life, 6-min-walk-test, responder index	
11	Yabroudi et al. (2023) [39]	34		24 sessions (approximately 2 months)	Knee	KOOS, NPRS; walking speed and 5-times chair stand test	Decreasing pain and improving physical function
12	Zorzi et al. (2007) [32]	31 (15 M, 16 F)	I-ONE IGEA	6 h per day for 90 days	Lower limbs	KOOS	Improving physical function
13	Ay et al. (2008) [40]	55 (15 M, 40 F)			Knee	VAS, Likert, WOMAC	Pain relief, range of motion (ROM)
14	Stubeyaz et al. (2005) [34]	34		30 min per session, twice a day for 3 weeks	Spine	VAS, NPDS	Pain, range of motion (ROM) and functional status
15	Thamsborg et al. (2005) [41]	83	Biofields Aps	2 h daily days per week for 6 weeks	Knee	WOMAC	Pain at all evaluations and stiffness
16	Pipitone et al. (2001) [42]	75	Medicur Devices	Three times a day	Knee	WOOMAC, Euro-QoL, SF-36	Reduction in overall pain
17	Danao Camara et al. (2001) [43]	167			Knee and Spine	VAS, modified Ritchie scale	Pain and physician global assessment

## 4. Discussion

The outcomes of the various studies investigating the efficacy of pulsed electromagnetic field (PEMF) therapy in osteoarthritis reveal promising results. Noteworthy improvements in pain scores were observed across the reviewed articles, with an overall mean treatment effect of −0.73 (95% CI −1.24 to −0.19) on the visual analog scale (VAS) and an effect size of −0.34 (95% CI −0.85 to 0.17) on the WOMAC score. Additionally, a substantial percentage of patients in the PEMF groups experienced positive changes, including a 26% reduction in medication usage and diverse improvements in clinical signs, quality of life, and pain intensity.

In a randomized controlled trial by Bagnato et al., utilizing the Bioelectronics Corporation’s PEMF device for 12 h daily over one month in knee osteoarthritis patients, a significant reduction in pain intensity emerged as the primary outcome [27]. This improvement was coupled with enhancements in quality of life and an increased pain threshold in response to pressure. Wuschech et al. explored Magcell Arthro in 57 participants with knee osteoarthritis, revealing significant reductions in pain, stiffness, and disability over an 18-day treatment period, highlighting the therapy’s tolerability and efficacy [31]. Additionally, a study protocol focusing on postmenopausal osteoporosis and lumbar osteoarthritis outlined various outcomes, such as BBS, TUG, and more [33]. Dündar et al. evaluated the utility of PEMF therapy in knee osteoarthritis, demonstrating its potential to reduce pain and introducing the use of YKL-40 for assessing disease severity [36]. Collectively, these studies contribute to the evolving understanding of PEMF therapy’s role in managing osteoarthritis, showcasing its diverse applications and potential for enhancing patient outcomes [Table 2].

### 4.1. Parameters

The comprehensive analysis of the reviewed studies provides valuable insights into the application of PEMF therapy in osteoarthritis, considering key parameters such as the total number of patients enrolled, the anatomical districts targeted, and the variations in PEMF devices. The diverse range of patient enrollment, with sample sizes ranging from 22 to 428 individuals, reflects efforts to explore PEMFs’ impact across different cohorts. Larger trials, such as that by Xiang et al., including 428 participants, contribute substantial statistical power to assessing the non-inferiority of PEMFs compared to COXIB medication in managing knee osteoarthritis [30] [Table 2]. The consistent focus on the knee joint as the primary anatomical district affected by osteoarthritis aligns with the prevalence of studies in knee-related outcomes, although valuable investigations extend to other areas such as the lumbar spine and the cervical spine.

### 4.2. Type of PEMF Device

The diversity in PEMF devices utilized, including those from the Bioelectronics Corporation and the Better Health Corporation, highlights technological variability, prompting further investigation into the optimal characteristics of PEMF devices for specific osteoarthritic conditions. These studies thoroughly explored the use of different PEMF devices currently available in commerce, juxtaposing them with older models that are no longer in use. The technological differences among various PEMF devices play a pivotal role in influencing the therapy’s efficacy. Each device, characterized by unique features such as the frequency and intensity of the magnetic field, contributes to the overall variability in treatment outcomes [Table 2]. The results emphasize that high-frequency PEMFs may have a greater impact on inflammation, blood circulation, and muscle tone, making them more suitable for osteoarthritis treatment. Taking a detailed approach to understanding this technological variability could potentially aid in the development of more precise and customized guidelines for the clinical application of PEMF therapy. Consequently, this has the potential to significantly improve the effectiveness and relevance of this innovative form of treatment for patients dealing with osteoarthritis.

### 4.3. Treatment Duration and Frequency

Analyzing the duration of treatment and outcomes across the reviewed studies provides valuable insights into the temporal aspects and effectiveness of PEMF therapy in osteoarthritis management. The duration of PEMF treatment varied significantly among the studies, ranging from 2 weeks to 90 days. Shorter-term interventions, such as the two-week trial by Ozgüçlü et al. [28] and the 18-day treatment in the study by Wuschech et al. [31], aimed to evaluate more immediate effects. Longer-term studies, like the 90-day intervention by Zorzi et al. [32], were designed to assess the sustainability and cumulative impact of PEMF therapy on functional recovery after arthroscopic surgery [Table 2]. Despite the diverse treatment durations, positive outcomes were observed in both cases. Short-term treatments showed a notable reduction in acute pain, while long-term interventions were effective in preventing exacerbations and promoting sustained benefits. It is also essential to focus on treatment frequency. The studies analyzed had treatment frequencies ranging from 10 min to 12 h per day, and it is evident that a higher treatment frequency showed more encouraging results.

### 4.4. Outcomes and Result Heterogeneity

The consistent focus on pain reduction as a primary outcome, measured using visual analog scales or disease-specific scales, indicates positive trends in PEMFs’ analgesic effects. Diverse secondary outcomes, including quality of life assessments, changes in analgesic and NSAID intake, and improvements in physical function, reflect the multifaceted impact of PEMF therapy on osteoarthritis.

In examining the heterogeneous results across the studies, it is crucial to elucidate the diversity in the outcomes of pulsed electromagnetic field (PEMF) therapy and provide a comprehensive overview of its effectiveness. The varying responses observed can be attributed to several factors, including differences in patient demographics, the anatomical areas targeted, and the technological nuances of the employed PEMF devices. This inherent heterogeneity underscores the dynamic nature of PEMF therapy in managing osteoarthritis.

In light of these findings, understanding the dynamic nature of PEMF therapy, considering technological nuances, patient demographics, and targeted anatomical areas, becomes crucial for optimizing its application and improving outcomes in osteoarthritis management. Moreover, providing specific recommendations on the optimal duration and frequency of PEMF therapy enhances the translational relevance of the study’s findings, guiding practitioners towards effective implementation for optimal patient outcomes.

### 4.5. Study Limitations

This review has identified several significant limitations that require careful consideration. One primary constraint is the limited number of studies included, particularly when attempting to comprehensively analyze outcomes, assess treatment duration and scrutinize the specific types of PEMF used. The lack of available data on the equipment used introduces uncertainty. The research was confined to only two electronic databases, namely PubMed and Google Scholar. While these are commonly utilized sources, there are additional databases to ensure comprehensive coverage of the literature, such as Embase, Scopus, or the Cochrane Library. Additionally, the diversity in outcomes analyzed, measured using different assessment scales adopted by various authors, poses a challenge to achieving a standardized evaluation.

Despite these challenges, a consistent relationship between PEMF therapy and positive impacts on pain, physical function and quality of life emerged across studies suggesting a robust trend. Another limitation is the maximum duration of PEMF treatment capped at 90 days with no assessed follow-up period. This temporal constraint limits the clinical relevance of the results, highlighting the need for long-term follow-up studies to explore the sustained effects of PEMF therapy. Understanding the durability and potential changes in outcomes over an extended period is crucial for establishing the treatment’s lasting efficacy and safety.

Furthermore, this review’s scope was confined to studies related to osteoarthritis, overlooking potential benefits in other orthopedic fields such as painful prostheses or tendinitis. Exploring the applicability and effectiveness of PEMF therapy in diverse orthopedic conditions could provide a more comprehensive understanding of its therapeutic potential. While acknowledging these limitations, the consistency observed in the effects of PEMF therapy, as evidenced by the meticulous evaluation of primary and secondary outcomes, underscores the robustness of the findings. The highly significant magnitude of the associations further reinforces the value of these results within the studied context. 

To address these limitations and contribute to the advancement of knowledge in this field, future research endeavors should prioritize larger sample sizes, detailed reporting of the types of PEMF devices used and standardized outcome measures. Long-term follow-up studies are imperative to unravel the sustained effects and potential long-term risks of PEMF therapy. Exploring its application beyond osteoarthritis, especially in painful prostheses or tendinitis, could uncover additional therapeutic avenues.

While our study provides valuable insights, addressing these limitations in future research will undoubtedly contribute to a more nuanced understanding of the true potential and limitations of PEMF therapy in orthopedic conditions.

## 5. Conclusions

The variable duration of PEMF therapy, coupled with consistent and diverse outcomes, highlights its potential as a comprehensive approach for managing osteoarthritis, addressing pain and broader aspects of patient well-being and disease progression. The aggregated analysis emphasizes the dynamic nature of research on PEMF therapy in osteoarthritis, considering patient demographics, anatomical areas and the technological nuances of employed PEMF devices. The aim of this systematic review was to evaluate the effectiveness of these innovative treatments on osteoarthritis and, given the highly encouraging results we have highlighted, this comprehensive understanding sets the stage for future research directions and potential advancements in optimizing PEMF therapies for various osteoarthritic manifestations.

## Figures and Tables

**Table 1:** Schematic representation of results divided according to the number of patients, the treatment duration, the anatomic district involved, and outcomes based on evaluation scales.

Results			
Total enrolled patients	1197	35.8% M	64.1% F
Treatment duration	15 days to 90 days (median 52.5)		
Anatomical district	77% knee osteoarthritis (71% KL 2–3°, 14.2% KL 0–2°)	20% spine osteoarthritis	
Decrease in the VAS scale	60 ± 11%		
Improvement WOMAC score	42% (95% CI −85 to 17)		

## Data Availability

All the data we analyzed and tables we compiled are available for any clarification.
